# The CXCL2/IL8/CXCR2 Pathway Is Relevant for Brain Tumor Malignancy and Endothelial Cell Function

**DOI:** 10.3390/ijms22052634

**Published:** 2021-03-05

**Authors:** Ruth M. Urbantat, Anne Blank, Irina Kremenetskaia, Peter Vajkoczy, Güliz Acker, Susan Brandenburg

**Affiliations:** 1 Department of Experimental Neurosurgery, Charité—Universitätsmedizin Berlin, Corporate Member of Freie Universität Berlin and Humboldt-Universität zu Berlin, 10117 Berlin, Germany; ruth-maria.urbantat@charite.de (R.M.U.); anne.blank@charite.de (A.B.); irina.kremenetskaia@charite.de (I.K.); gueliz.acker@charite.de (G.A.); susan.brandenburg@charite.de (S.B.); 2 Department of Neurosurgery, Charité—Universitätsmedizin Berlin, Corporate Member of Freie Universität Berlin and Humboldt-Universität zu Berlin, 10117 Berlin, Germany; 3 Berlin Institute of Health, 10178 Berlin, Germany

**Keywords:** tumor angiogenesis, chemokines, glioblastoma, SB225002, HBMEC

## Abstract

We aimed to evaluate the angiogenic capacity of CXCL2 and IL8 affecting human endothelial cells to clarify their potential role in glioblastoma (GBM) angiogenesis. Human GBM samples and controls were stained for proangiogenic factors. Survival curves and molecule correlations were obtained from the TCGA (The Cancer Genome Atlas) database. Moreover, proliferative, migratory and angiogenic activity of peripheral (HUVEC) and brain specific (HBMEC) primary human endothelial cells were investigated including blockage of CXCR2 signaling with SB225502. Gene expression analyses of angiogenic molecules from endothelial cells were performed. Overexpression of VEGF and CXCL2 was observed in GBM patients and associated with a survival disadvantage. Molecules of the *VEGF* pathway correlated but no relation for *CXCR1/2* and *CXCL2/IL8* was found. Interestingly, receptors of endothelial cells were not induced by addition of proangiogenic factors in vitro. Proliferation and migration of HUVEC were increased by VEGF, CXCL2 as well as IL8. Their sprouting was enhanced through VEGF and CXCL2, while IL8 showed no effect. In contrast, brain endothelial cells reacted to all proangiogenic molecules. Additionally, treatment with a CXCR2 antagonist led to reduced chemokinesis and sprouting of endothelial cells. We demonstrate the impact of CXCR2 signaling on endothelial cells supporting an impact of this pathway in angiogenesis of glioblastoma.

## 1. Introduction

Glioblastomas (GBM) are characterized by high invasiveness as well as increased angiogenesis [1]. Despite extensive research and combined therapy approaches including surgery, radiotherapy and chemotherapeutics, the median survival is only 12–15 months [2].

VEGF is one of the most important angiogenic factors for tumor angiogenesis [3] and is overexpressed in GBM tissues [4]. Thus, VEGF was identified as a feasible target for glioma therapy [5,6]. However, targeting the enhanced angiogenesis by inhibitors of VEGF or its receptors, VEGFR1 and VEGFR2, did not lead to a significantly improved survival [7,8,9], whereby development of resistance to the anti-angiogenic agents is often observed [10,11]. Consequently, mechanisms of resistance formation in response to anti-angiogenic approaches got into the focus of further research.

The alternative proangiogenic factors CXCL2 and IL8, which act through CXCR2 or CXCR1, seem to be promising therapeutic targets due to their expression in glioma cell lines and mouse models [12,13,14,15]. The G-protein-coupled receptor of both molecules, CXCR2, is one of the most important receptors mediating angiogenesis through chemokines. CXCR2 is expressed by a variety of cell types, e.g., endothelial cells, glioma cells, T lymphocytes, mast cells and myeloid cells [16,17,18,19,20]. Thus far, the effect of the IL8/CXCR2 pathway on human tumor cells was studied in vitro and immunodeficient rodent models [18]. There, an upregulation of IL8 and CXCR2 was demonstrated within the tumor after anti-angiogenic treatment with VEGF-pathway inhibitors and a decrease of tumor-derived endothelial-like cells as well as tumor stem cells after application of the CXCR2-antagoist SB225002 [18]. In parallel, we showed that this antagonist led to a lower vessel density and decreased infiltration of microglia/macrophages with a consecutive tumor volume reduction using an immunocompetent GBM rodent model focusing on the CXCL2/CXCR2 signaling pathway due to lacking the IL8 expression in mice [21].

In previous studies, the impact of IL8 on umbilical vein endothelial cells (HUVEC) has been investigated in vitro [22,23]. CXCL2 is barely evaluated and little is known about the efficacy of CXCL2 and IL8 compared to the angiogenic potential of VEGF. Besides, endothelial cells from various parts of the body could behave differently showing cell-origin specific reactivity to angiogenic stimuli [24,25]. However, data concerning primary brain endothelial cells (HBMEC) to analyze the impact of proangiogenic molecules relevant for brain tumor vascularization are lacking so far. In our study, we defined expression and significance of VEGF-alternative proangiogenic factors like CXCL2 and IL8 in human glioblastoma tissues. Furthermore, we performed various in vitro assays to investigate the angiogenic capacity of these molecules in comparison to VEGF. Our results showed that CXCL2 and IL8 are potent proangiogenic factors. Thus, the CXCL2/IL8/CXCR2 axis could be a relevant pathway to circumvent the disturbance of the VEGF/VEGFR signaling in glioblastomas to maintain tumor angiogenesis.

## 2. Results

### 2.1. Human GBM Tissues Showed Expression of VEGF as well as CXCL2 and IL8

**Figure 1:**
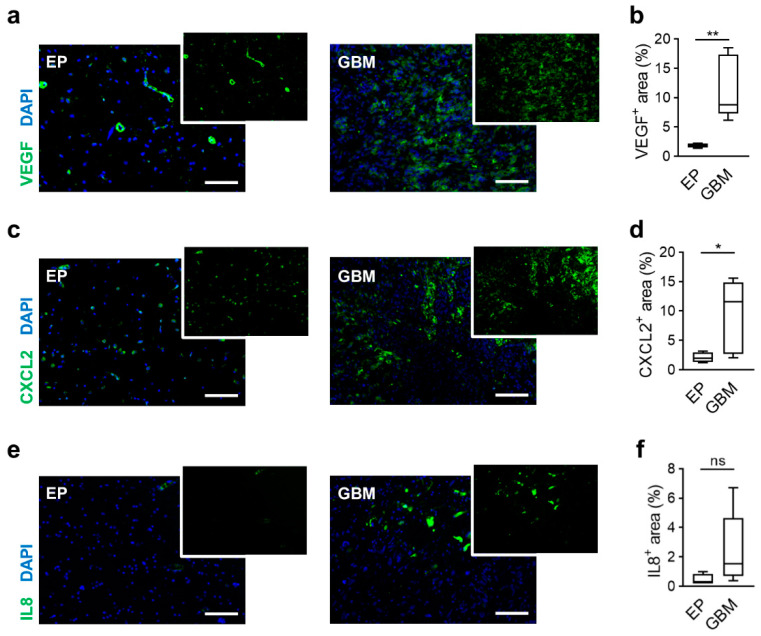
Overexpression of proangiogenic factors in human GBM. (**a**,**c**,**e**) Tissue sections of epilepsy (EP) and glioblastoma (GBM) patients were stained for proangiogenic factors like VEGF (**a**), CXCL2 (**b**) and IL8 (**c**). green: indicated molecule, blue: DAPI (nuclei). Scale bars 100 µm. (**b**,**d**,**f**) Graphs depict calculation of the stained area of VEGF (**b**), CXCL2 (**d**) and IL8 (**f**). * *p* < 0.05, ** *p* < 0.01, ns = not significant. Student’s *t*-test. *n* = 4 in EP and *n* = 8 in GBM.

Angiogenesis is a hallmark for the progression of glioma. VEGF was identified as one of the key mediators of angiogenesis showing overexpression in murine and human glioblastoma tissues [26,27]. Accordingly, we observed a widespread expression of VEGF in human glioma samples directly collected after surgery, while control tissues of epilepsy patients depicted only local VEGF^+^ staining (Figure 1a). The area of VEGF expression varied between GBM patients (median: 8.78%, range: 6.17–18.5%; Figure 1b). Besides, VEGF-alternative proangiogenic factors such as CXCL2 and IL8 were observed within tumors [28,29,30]. In human GBM specimens, we found pronounced CXCL2 staining in contrast to control tissue where single cells expressed this molecule (Figure 1c). Even though variance between glioma samples was high (median: 11.6%, range: 2.05–15.6%), we detected a significant increase of CXCL2 expression (Figure 1d). Furthermore, the median of CXCL2 expression was higher than the median of VEGF, implicating an extensive production of CXCL2 within glioma tissues. IL8 also showed almost no staining within epilepsy tissues (Figure 1e), while we observed in GBM specimens a tendency of higher IL8 expression but with a wide interindividual range (median: 1.55%, range: 0.38–6.71%; Figure 1f). Overall, we found expression of VEGF and the alternative proangiogenic factors CXCL2 and IL8 in human GBM samples, supporting our findings about the importance of the CXCR2 signaling pathway for tumor progression [21].

### 2.2. Gene Expression of Proangiogenic Factors Correlates with Survival of Human GBM Patients

**Figure 2:**
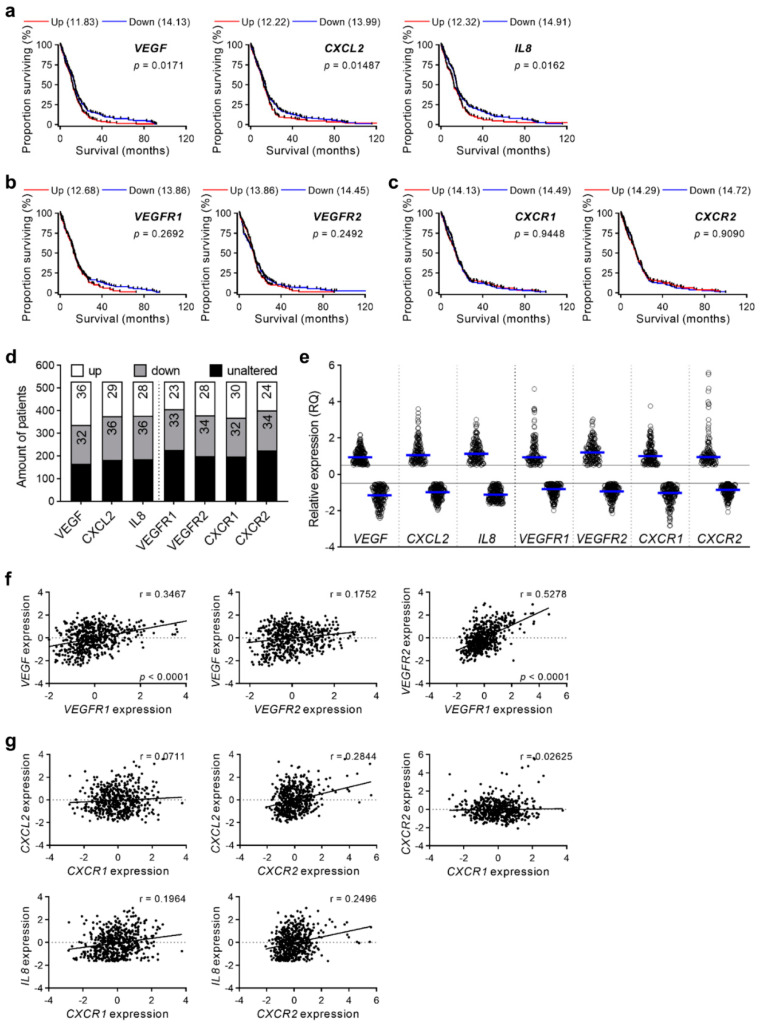
TCGA database analyses related to proangiogenic factors and their respective receptors. (**a**–**c**) TCGA database was used to prepare survival curves of GBM patients concerning proangiogenic factors *VEGF, CXCL2* and *IL8* (**a**), and the receptors *VEGFR1*, *VEGFR2* (**b**) as well as *CXCR1* and *CXCR2* (**c**). Patients were clustered in groups with upregulation (Up, z-score > +0.5) and downregulation (Down, z-score < −0.5) of molecules. VEGF (Up: *n* = 166, Down: *n* = 151), CXCL2 (Up: *n* = 134, Down: *n* = 169), IL8 (Up: *n* = 133, Down: *n* = 160), VEGFR1 (Up: *n* = 99, Down: *n* = 160), VEGFR2 (Up: *n* = 121, Down: *n* = 156), CXCR1 (Up: *n* = 122, Down: *n* = 152), CXCR2 (Up: *n* = 113, Down: *n* = 153). Median survival (months) of groups as well as p-values are indicated. Log-rank (Mantel-Cox) test. (**d**,**e**) Expression data of GBM patients regarding proangiogenic molecules and their receptors from the TCGA database were analyzed. Percentage of patients with unaltered, upregulated, and downregulated molecule and receptor expression were calculated (**d**). Graph represents relative expression of analyzed genes. blue line: mean value of up- and downregulated samples, each dot illustrate one patient sample (**e**). z-score ± 0.5, *n* = 528. (**f**,**g**) Correlation of molecule and the respective receptor expression was analyzed. VEGF related genes (**f**) and CXCR2 pathway molecules (**g**) were correlated. Linear regression analyses were performed (Spearman correlation). *r* and significant *p* values are indicated; *p* ≤ 0.05 was considered statistically significant. *n* = 528.

To evaluate the clinical importance of the shown overexpression of different proangiogenic molecules, the TCGA database was used to plot Kaplan–Meier survival curves. Here, overexpression of the classical angiogenic pathway molecule *VEGF* showed a significantly shortened overall survival. The VEGF-alternative proangiogenic factors such as *CXCL2* and *IL8* depicted similar outcomes, resulting in a worse overall survival of patients with upregulated molecule expression (Figure 2a). Furthermore, we investigated the impact of respective receptors *VEGFR1* and *VEGFR2* (Figure 2b) as well as *CXCR1* and *CXCR2* (Figure 2c) on survival, but no differences were detected between the patient subsets stratified by up- and downregulation. When we analyzed the gene expression of the 528 patients from the TCGA database, we found the strongest regulation of *VEGF* among these molecules, whereby about one third of samples showed overexpression (Figure 2d). Interestingly, *CXCL2* and *IL8* were similarly robustly regulated, but revealed fewer specimens with a higher expression. *VEGFR1* and *CXCR2* were the least effected molecules showing overexpression in only up to 24% of patients.

Considering the individual expression, we elucidated that some specimens displayed stronger expression of *CXCL2, IL8* and *CXCR2* than VEGF/VEGFR pathway molecules (Figure 2e), suggesting relevance of these VEGF-alternative proangiogenic molecules in certain cases. Interestingly, these alternative proangiogenic molecules showed only low correlation to their receptors. *VEGF* correlated with *VEGFR1* and both *VEGF*-receptors were linked to expression of each other (Figure 2f) while *CXCL2* and *IL8* did not exhibit any dependency to the *CXCR1* and *CXCR2* expression (Figure 2g).

### 2.3. RNA Expression Profiles of Proangiogenic Pathway Receptors in Human Endothelial Cells Do Not Differ after Stimulation with Respective Ligands In Vitro

**Figure 3:**
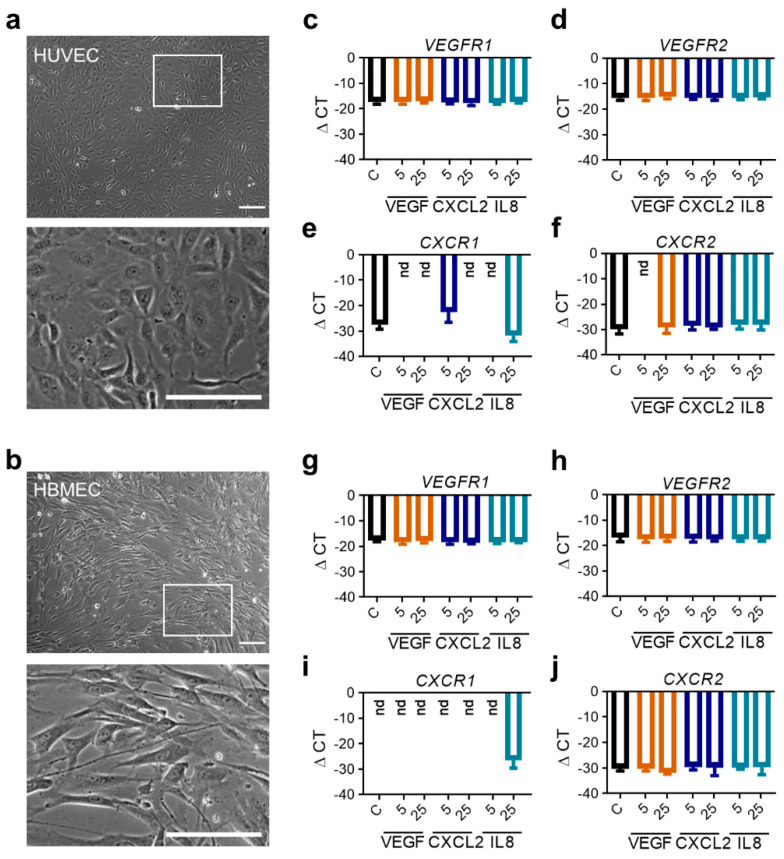
RNA expression analyses of angiogenic receptors in human endothelial cells following stimulation with angiogenic molecules. (**a**,**b**) Morphology of HUVEC (**a**) and HBMEC (**b**) in culture taken by phase contrast microscopy. Scale bars 150 µm, squares: identified magnified areas. (**c**–**j**) HUVEC (**c**–**f**) and HBMEC (**g**–**j**) were stimulated with VEGF, CXCL2 or IL8 in indicated concentrations for 24 h. Analysis of mRNA expression regarding the indicated proangiogenic receptors are depicted (*n* = 9/condition out of three independent experiments). nd: not detected.

To investigate the relevance of the detected proangiogenic molecules CXCL2 and IL8 within glioma tissues on endothelial cell function, we performed various in vitro assays with endothelial cells from the periphery (Human Umbilical Vein Endothelial Cells, HUVEC) and the brain (Human Brain Microvascular Endothelial Cells, HBMEC). Both low passaged primary endothelial cell populations were grown in culture. Interestingly, HUVEC and HBMEC showed different morphology (Figure 3a,b). HUVEC showed a plump and compact phenotype whereas HBMEC cells were significantly longer and thinner. First, we evaluated whether HUVEC and HBMEC express the receptors of VEGF, *VEGFR1* and *VEGFR2* [31,32,33], as well as the receptors for CXCL2 and IL8, *CXCR1* and *CXCR2* [19,28,34,35] by real-time PCR. In both cell populations the basal expression of *VEGF*-receptors (Figure 3c,d,g,h) and *CXCR2* (Figure 3f,j) was comparable, whereby *CXCR2* was expressed to lesser extent than *VEGFR1/2. CXCR1* could only be detected in HUVEC (Figure 3e,i). Furthermore, we stimulated HUVEC as well as HBMEC with the ligands of the angiogenic receptors for 24 h in vitro and analyzed their gene expression. Interestingly, no significant changes of all receptors in both endothelial cell types were observed when stimulated by VEGF, CXCL2 or IL8 in different concentrations (Figure 3c–j). To examine whether receptor expression depends on the time of stimulation, we additionally cultivated cells for 4 and 18 h with indicated angiogenic molecules. However, results were comparable to 24 h of molecule treatment (data not shown). Thus, despite availability of ligands, the receptor gene expression of HUVEC and HBMEC could not be induced in vitro.

### 2.4. Mobilization of Endothelial Cells by the Classical Proangiogenic Factor VEGF in Comparison to the Alternative Molecules CXCL2 and IL8

**Figure 4:**
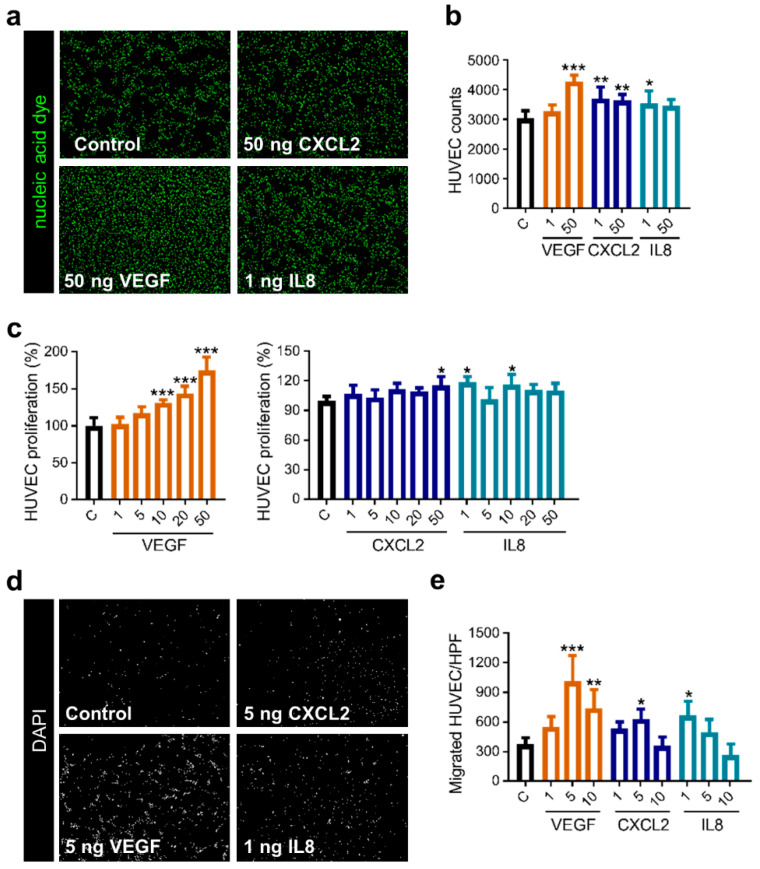
Stimulation with CXCL2 and IL8 promotes proliferation and migration of HUVEC. (**a**–**c**) CyQUANT assay was performed with HUVEC, adding recombinant proteins as indicated (ng/mL). Representative images of the nucleic acid stain are depicted (**a**). Graph shows counted cells from one representative experiment of three independent experiments with similar results (*n* = 4–5 wells/condition) (**b**). Graphs illustrate calculation of proliferation measured by fluorescence intensity from one representative experiment of three independent experiments with similar results (*n* = 4–5 wells/condition) (**c**). * *p* < 0.05, ** *p* < 0.01, *** *p* < 0.001; one-way ANOVA. (**d**,**e**) Migration of HUVEC was accessed using a Boyden chamber assay in reaction to VEGF, CXCL2 and IL8 in different concentrations (ng/mL). Images depict one representative high-power field (HPF; magnification: 200×) for the respective condition (**d**). Graph represents one experiment of four independent experiments with similar results (*n* = 3–4 membranes/condition) (**e**). * *p* < 0.05, ** *p* < 0.01, *** *p* < 0.001; one-way ANOVA.

The positive effect of VEGF on endothelial cells is well known, while influence by CXCL2 and IL8 is analyzed to a lesser extent [36,37]. Hence, we evaluated the proliferative and migratory activity of human endothelial cells in reaction to these molecules. Counting cells following stimulation with angiogenic factors revealed that VEGF as well as CXCL2 and IL8 were able to induce proliferation of HUVEC (Figure 4a,b). Colorimetric analysis showed a dose-dependent increase in proliferative activity if rising concentrations of VEGF were added, while CXCL2 and IL8 resulted in enhanced proliferation by using specific molecule concentrations (Figure 4c). Additionally, investigation of the endothelial cell migration revealed that both factors significantly improved the motility of HUVEC in a dose-dependent manner, even though CXCL2 and IL8 did not reach the high level of migratory response as mediated by VEGF (Figure 4d,e). Consequently, CXCL2 and IL8 can induce proliferation and mobilization of primary endothelial cells but with lesser potency than VEGF.

### 2.5. CXCL2 and IL8 Show Enhanced Angiogenic Capacity on Brain Endothelial Cells Compared to VEGF

**Figure 5:**
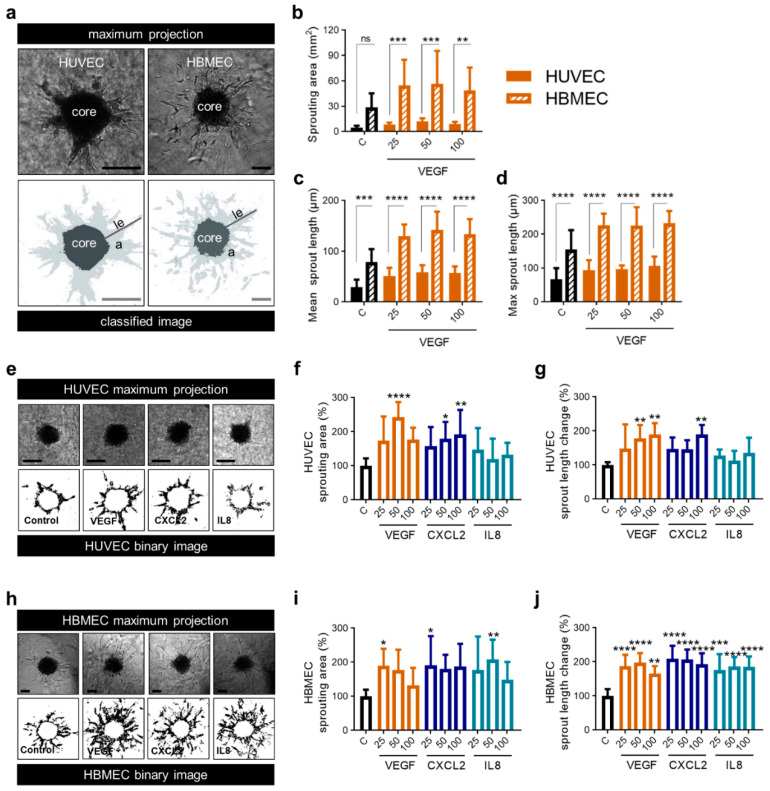
CXCL2 and IL8 show enhanced angiogenic capacity on brain endothelial cells compared to VEGF. (**a**–**d**) 3D Sprouting assay of HUVEC and HBMEC spheroids was performed in response to the treatment with VEGF as indicated (25, 50 and 100 ng/mL). Representative images show the maximal projection and the binary image of spheroids Scale bars 100 µm. le: length, a: area (light grey) (**a**). Sprouting area (**b**), mean sprout length (**c**) and maximum sprout length (**d**) of HUVEC and HBMEC spheroids were calculated. Graphs represents multiple experiments with similar results (*n* = 8–12 spheroids/condition). (**e**–**g**) 3D Sprouting assay of HUVEC spheroids was performed in response to the treatment with VEGF, CXCL2 and IL8 as indicated (25, 50 and 100 ng/mL). Representative images show the maximal projection and the binary image of spheroids. Scale bars 100 µm (**e**). Sprouting area (**f**) and mean sprout length change (**g**) were calculated. Data represent multiple experiments with similar results (*n* = 6–10 spheroids/condition). (**h**–**j**) 3D Sprouting assay of HBMEC spheroids was performed in response to the treatment with VEGF, CXCL2 or IL8 as indicated (25, 50 and 100 ng/mL). Representative images show the maximal projection and the binary image of spheroids. Scale bars 100 µm (**h**). Sprouting area (**i**) and mean sprout length change (**j**) were calculated. Data represent multiple experiments with similar results (*n* = 7–12 spheroids/condition). (**a**–**j**) Medium containing 0.1% FCS was used as control (C). ns = not significant, * *p* < 0.05, ** *p <* 0.01, *** *p* < 0.001, **** *p* < 0.0001; one-way ANOVA (Bonferroni correction).

To further characterize the functional effect of proangiogenic molecules on endothelial cell populations, we performed an in vitro angiogenesis assay using spheroids of HUVEC as well as brain endothelial cells. Sprouting assays for HUVEC are established [38], while corresponding assays with primary brain endothelial cells were lacking. However, investigating the effect of VEGF, CXCL2 and IL8 on HBMEC was of special interest because these cells may react differently than the well-established HUVEC. Therefore, we first set up a novel protocol for the 3D angiogenesis assay with HBMEC. We tested different cell numbers, embedding conditions and collagen concentrations. In comparison to HUVEC, we had to use twice as many cells (500 cells/spheroid vs. 1000 cells/spheroid) and a higher concentration of Methocel (0.6% vs. 1.2%) to obtain stable HBMEC spheroids. Other parameters were unchanged, guaranteeing the comparability of experiments. Following establishment, we observed robust sprouting of HBMEC which differed from sprouting of HUVEC spheroids (Figure 5a). HBMEC showed higher basic sprouting parameters than HUVEC, e.g., total sprouting area (Figure 5b) and sprout length (Figure 5c,d). Additionally, after stimulation with VEGF in various concentrations the sprouting area of HBMEC was fivefold higher (Figure 5b) and mean sprout length 2.5-fold longer in comparison to HUVEC (Figure 5c), thus implicating a strong angiogenic activity of HBMEC. Compared to the control, all concentrations of VEGF on HBMEC as well as 25 and 100 ng/mL on HUVEC led to a two-fold increase of the sprouting area whereas 50 ng/mL VEGF led to a threefold increase of the sprouting area on HUVEC (Figure 5b). The alteration in mean sprout length compared to the control was similar between HUVEC and HBMEC (Figure 5c).

Treatment of HUVEC and HBMEC with VEGF, CXCL2 and IL8 led to the induction of sprouting. Interestingly, HUVEC spheroids only showed significant alterations if VEGF and CXCL2 were applied in higher concentrations (Figure 5e–g) while angiogenesis assay of HBMEC revealed enhanced sprouting after adding VEGF, CXCL2 as well as IL8 even at low doses (Figure 5h–j). In this approach, VEGF, CXCL2 and IL8 had comparable angiogenic capacity on HBMEC whereas HUVEC were less affected by CXCL2 and IL8.

### 2.6. Blockade of CXCR2 Reduces Chemokinesis and Angiogenesis In Vitro

As we verified the angiogenic potential of CXCL2 and IL8, implying an involvement of the CXCR2 axis in tumor angiogenesis, we aimed to investigate the effect of CXCR2 antagonist SB225002 on primary human endothelial cells. SB225002 has already been shown to have an impact on vessel density in a mouse model in vivo as well as to decrease tumor endothelial cells and infiltration of microglia/macrophages [21]. These cells are an essential source of proangiogenic molecules like CXCL2 in mice and human GBM [14,39,40].

**Figure 6:**
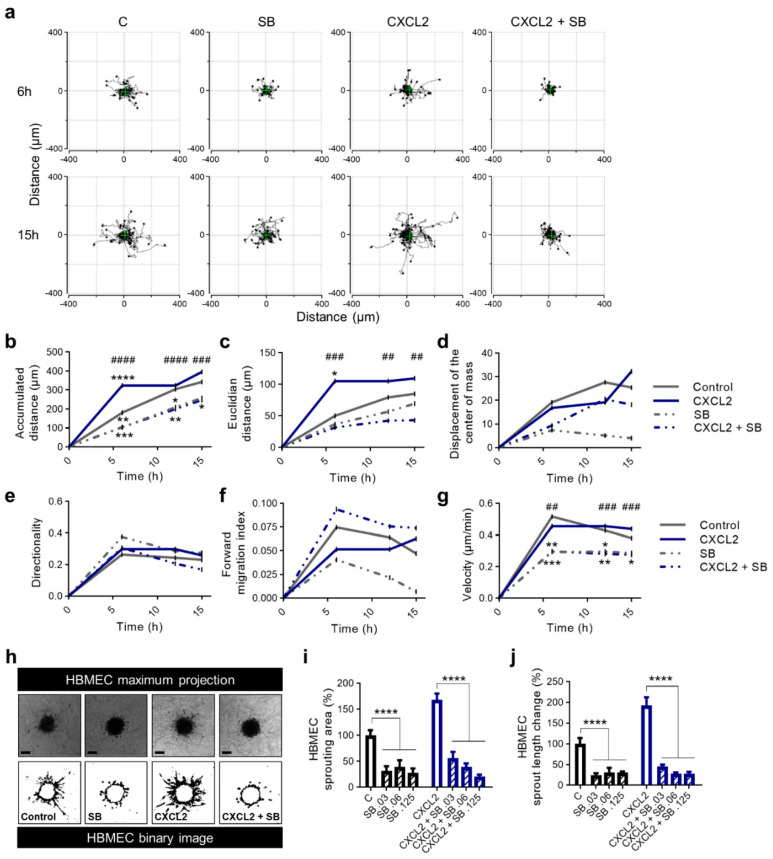
SB225002 reduces chemokinesis and sprouting ability of primary human endothelial cells. (**a**–**g**) HUVEC were seeded into µ-slides. CXCL2 (10 ng/mL), SB225002 (SB), a combination of CXCL2 + SB225002 and 0.1% FCS in ECBM2 medium as control were added to create a chemokine gradient. The cells were imaged over 15 h by time-lapse microscopy and analyzed after 6, 12 and 15 h. Representative trajectory plots after 6 and 15 h are depicted. The green cross represents the center of mass (**a**). Quantitative data of accumulated distance (**b**), Euclidian distance (**c**), displacement of the center of mass (**d**), directionality (**e**), forward migration index (**f**) and velocity (**g**) are shown. Rayleigh test *p* values were not significant for any condition indicating that the distribution of the cell end points was homogenous. Graphs show the mean values of all surviving tracked cells of one representative experiment. * indicating the level of significance compared to the control group; ^#^ indicating the level of significance comparing CXCL2 with CXCL2 + SB (**b**–**g**). (**h**–**j**) 3D Sprouting assay of HUVEC spheroids was performed in response to the treatment with CXCL2 (25 ng/mL) and SB225002 (0.03 µM, 0.06 µM, 0.125 µM) and in combination as indicated. Representative images show the maximal projection and the binary image of spheroids. Scale bars 100 µm (**h**). Sprouting area (**i**) and mean sprout length change (**j**) were calculated. Graphs represent multiple experiments with similar results (*n* = 3-4 spheroids/condition). * *p* < 0.05, ** *p* < 0.01, *** *p* < 0.001, **** *p* < 0.0001; ^##^ *p* < 0.01, ^###^ *p* < 0.001, ^####^ *p* < 0.0001; one-way ANOVA (Bonferroni correction).

First, we realized a chemotaxis assay using HUVEC in µ-Slide chambers to track the motility of single cells. We were able to demonstrate that SB225002 inhibited the action of endothelial cells with and without simulated overexpression of CXCL2 (Figure 6a). Overall, the strongest changes of all parameters were observed within 6 h (Figure 6b–g). Here, the accumulated distance, Euclidian distance and velocity reached a plateau and the directionality and forward migration index (FMI) decreased. Application of the CXCR2 antagonist led to changes of chemokinetic parameters, including reduction in accumulated distance (Figure 6b), Euclidian distance (Figure 6c) and velocity (Figure 6g). The equally pronounced effect of SB225002 on CXCL2 treated and non-treated cells of the control group suggest a high efficacy of the CXCR2 antagonist. However, there was no significant increase in the displacement of the center of mass (Figure 6d) nor the FMI (Figure 6f) towards CXCL2 or SB225002. Therefore, no chemotactic effect of these molecules in this experimental setup could be observed. Furthermore, *p* values for the Rayleigh test were not significant after 15 h (data not shown) indicating a homogenous distribution of cells even at the end point of experiments.

Second, we analyzed the application of SB225002 on HBMEC in the angiogenesis assay leading to detached cells that were not detected without the antagonist (Figure 6h). We found a significant reduction of the sprouting area (Figure 6i) and sprout length (Figure 6j) after treatment of HBMEC with various concentrations of SB225002 during stimulation with CXCL2. Once more underlining the efficacy of SB225002 in an environment of simulated overexpression of CXCL2. Nevertheless, after treatment with SB225002 the control group showed a decrease in the analyzed parameters as well.

## 3. Discussion

The CXCR2/IL8/CXCL2 signaling pathway has been shown to be crucial in GBM progression and development of resistance [13,18,30,41]. In this study, we demonstrated the impact of CXCL2 and IL8 on proliferation, migration and sprouting of human endothelial cells. We showed for the first time that these alternative proangiogenic molecules are efficient to stimulate human endothelial cell function in vitro similar to VEGF. Furthermore, we observed different effects on primary peripheral and brain endothelial cells. To highlight the importance of CXCL2 and IL8, their overexpression was defined in human glioblastoma specimens and a survival disadvantage was determined using TCGA database. Regarding possible therapeutic options, the impact of a CXCR2 antagonist was examined, revealing reduced chemokinesis and angiogenesis of human endothelial cells in general and during mimicked overexpression of CXCL2.

New therapeutical approaches are demanded due to the rapid development of resistance to the standard therapy, the lack of adequate long-term treatment and the poor overall survival in GBM. Angiogenesis as a hallmark of cancer is a crucial target in GBM treatment [42,43,44]. However, therapeutic approaches targeting the VEGF/VEGFR pathway in GBM did not lead to a prolonged overall survival due to a range of resistance mechanisms [10,11,18,45]. While CXCL2, IL8 and their respective receptor CXCR2 seem to contribute to angiogenesis and tumorigenesis in GBM [13,18], there is also evidence suggesting an important role of those molecules in circumvention of anti-angiogenic therapy in gliomas. Thus, IL8 and CXCR2 were upregulated in vitro and in vivo after treatment with VEGF pathway inhibitors and could contribute to development of therapy resistance [18,41]. Furthermore, a recent study with a small number of participants implied that GBM patients with a combined overexpression of *CXCR2* and *IL8* have a reduced overall survival and progression free survival [18]. Our analyses confirmed this observation, we demonstrated that even the sole overexpression of *IL8* and *CXCL2* predicted a shortened overall survival using the TCGA database. Nearly 30% of GBM patients showed an upregulation of *IL8* and *CXCL2*, indicating the importance of alternative proangiogenic pathways. In addition, Yang et al. also reported a correlation of high enhanced expression of the common receptor CXCR2 with malignancy and recurrence of gliomas [13]. Interestingly, we could not determine an impact on the overall survival amongst the TCGA patient cohort for the proangiogenic receptors. However, we showed a correlation of *VEGFR1* expression with *VEGF* while *VEGFR2*, *CXCR1*, *CXCR2* were not associated with the expression of their respective ligands. Our previous study including the GBM patient cohort of the TCGA database demonstrated a correlation of *IL8* and *CXCL2*, whereas *VEGF* gene expression only correlated with IL8 expression and not to CXCL2 [40], indicating independent pathways. Current data on autocrine VEGFR signaling in GBM are conflicting. While some studies proposed that VEGFR2 signaling promoted cell invasion and tumor growth [46,47], others reported an inhibition of invasiveness [48]. The lack of influence of the VEGFR overexpression on the overall survival could be due to these divergent functions.

While the effect of CXCL2 on endothelial cells is only marginally investigated, the impact of IL8 has already been extensively evaluated. IL8 enhances chemotaxis [23,49], proliferation, cell survival and angiogenesis [22,23] on various endothelial cell types. The angiogenic capacity of IL8 is based on the expression of CXCR1 and CXCR2 on endothelial cells [28], e.g., HUVEC, microvascular and brain endothelial cells [19,22,28,34]. Most in vitro studies of the CXCR2 signaling pathway have been conducted on HUVEC [22,23]. However, there are indications about differences on receptor expression between endothelial cell types [50]. Interestingly, we detected no variations in basal expression of angiogenic receptors between HUVEC and HBMEC, although HBMEC were described to be special to other vascular endothelial cells [51,52,53] based on their blood–brain barrier characteristics [54]. Nevertheless, for the first time, we demonstrated that HUVEC and HBMEC reacted differently to CXCL2 and IL8. In our newly established 3D spheroid-based angiogenesis model with HBMEC, CXCL2 and IL8 were highly effective and showed strong angiogenic activity. Their effect on HBMEC was comparable to VEGF, whereas their impact on HUVEC was inferior to VEGF. Additionally, our data demonstrated that the analyzed alternative proangiogenic factors had a pronounced impact on the proliferation as well migration of primary human endothelial cells similar to VEGF. Other studies support our findings of the importance of CXCR2 signaling in angiogenesis, proliferation and development of resistance in GBM while mainly focusing on IL8 [13,18,28,29,55]. Our results implicate, for the first time, variable effects of the alternative angiogenic factors depending on the origin of endothelial cells as an additional opportunity to use the CXCR2 axis in the specialized compartment of the CNS. This underlines the importance of the CXCR2 axis bypassing the VEGF-mediated angiogenesis pathways in the development of therapy resistance in human glioblastoma.

The gene expression of *VEGFR1/2* and *CXCR1/2* of HUVEC and HBMEC did not change after stimulation with VEGF, CXCL2 and IL8 in vitro. Whereas some studies have shown that treatment with VEGF decreased the protein level and increased the mRNA level of VEGFR1 under physiological conditions [56], our data did not support those findings. Nevertheless, different studies have found that VEGF did not increase expression of its receptors [57,58]. Rather than VEGFR1 the expression of soluble VEGFR1, an anti-angiogenic factor and splice variant of VEGFR1 mediated through VEGFR2-MEK-PKC signaling in endothelial cells was upregulated through VEGF [58]. Thus, the response of HUVEC and HBMEC in vitro initiated by VEGF, CXCL2 and IL8 despite the non-altered gene expression could be explained by alternative splicing, posttranscriptional alterations or an increased downstream signaling.

The strong effect of the CXCR2 antagonist SB225002 on angiogenesis and the chemokinetic behavior of endothelial cells suggests a high efficacy which corroborates our previous findings in a murine GBM model [21]. There we found a decreased vessel density, lower infiltration of tumor-associated microglia/macrophages and smaller tumor volumes after treatment with SB225002 [21]. Nevertheless, cells of the control group also reacted to the CXCR2 antagonist. This effect could be explained by the previously described anti-mitotic and anti-proliferative effects of the antagonist [59,60]. Petreacea et al. showed that the activation of CXCR2 in human microvascular endothelial cells could result in the co-activation of VEGFR2, independent of VEGF [61]. Furthermore, there is evidence for crosstalk between VEGF and the CXCL2/IL8 signaling pathway, mediated by anti-apoptotic BCL-XL and BCL-2 [62,63,64]. There, BCL-XL upregulates VEGF via MAPK/ERK signaling pathway. Through VEGFR2 signaling, VEGF leads to the expression of BCL-2. The upregulation of BCL-2 was described to lead to expression of IL8 in human endothelial cells [63]. This crosstalk could potentially be reversed by the application of SB225002. Therefore, it is possible that the inhibition of CXCR2 could indirectly impair VEGFR signaling via those pathways which in combination with the anti-mitotic anti-proliferative effects could account for the reaction of cells within the control group.

## 4. Materials and Methods

### 4.1. Human Specimens

Brain tissue samples of 12 patients were collected during therapeutic surgical treatment (Department of Neurosurgery, Charité-Universitätsmedizin Berlin, Germany) from 2013 to 2014. Specimens of four epilepsy patients who underwent temporal pole resection (control group), and GBM patients (8 cases) were evaluated. Independent neuropathologists verified pathological diagnosis by standard histologic markers. Patients’ characteristics are shown in Table 1. Approval of the Ethical Committee of Charité-Universitätsmedizin Berlin was received (application number: EA4/065/13; 12 June 2013) and all analyses were carried out following the defined obligations of scientific working with patient material. Informed consent was obtained from all subjects.

### 4.2. Analyses of TCGA Database

Gene expression data were obtained from the GBM patient dataset available through The Cancer Genome Atlas (TCGA; Affymetrix U133A) [65,66]. Patients were stratified into “up” versus “down” subsets based on gene expression (mean > +0.5 and mean < −0.5) to create Kaplan–Meier survival curves. Furthermore, the gene expression data of 528 patients were used to depict correlation analyses.

### 4.3. Immunofluorescence Staining

Directly after surgery, tissues were embedded in 4% PFA for 24 h, and subsequently dehydrated in a serial dilution with rising concentrations of sucrose. Afterwards, samples were frozen in liquid nitrogen. Frozen sections of 10 µm were prepared and treated with Autofluorescence Eliminator Reagent (Merck/Millipore, Darmstadt, Germany) following the instructions of the manufacturer.

Sections for staining of proangiogenic molecules CXCL2 and VEGF were fixed with ice-cooled methanol for 10 min. All slices were blocked in 1% Casein/PBS for 30 min, and subsequently stained with primary antibodies (CXCL2: AbD Serotec, Puchheim, Germany, AHP773, 1/100; VEGF: Abcam, Cambridge, UK, ab46154, 1/100) for 2 h at room temperature. After washing slices for 20 min in 0.5% Casein/PBS, secondary antibodies (FITC-conjugated anti-rabbit IgG, Dianova, Hamburg, Germany, 1/200) were applied. After incubation for 1.5 h at room temperature, sections were washed with PBS and water for 15 min each. DAPI-containing mounting medium (Dianova, Hamburg, Germany) was used to stain nuclei.

For IL8 (Abcam, Cambridge, UK ab10769, 1/50) staining, slices were treated with Antigen Retrieval Reagent (ARS) UNIVERSAL 10X (R&D Systems, Wiesbaden, Germany) as recommended in the manufacturer’s instructions after Autofluorescence Eliminator Reagent. Blocking was carried out using 1% Casein/PBS + 0.1% Triton-X100 for 30 min at room temperature directly after processing ARS protocol. Slices were incubated with the primary antibody overnight at 4 °C. After several wash steps, the secondary antibody (FITC-conjugated anti-goat IgG, Dianova, Hamburg, Germany, 1/200) was added. After 1.5 h incubation at room temperature, slices were washed and covered with DAPI-containing mounting medium. Images were acquired by Zeiss Axio Observer Z1 fluorescence microscope (Zeiss MicroImaging GmbH, Jena, Germany) at room temperature. ImageJ 1.53c (http://imagej.nih.gov/ij, NIH, USA) was used to analyze images. 12–16 images for each patient at three different brain tissue areas were analyzed.

### 4.4. Cultivation of Human Endothelial Cells

HUVEC were obtained from Promocell and cultivated in ECGM2 (Promocell, Heidelberg, Germany) containing supplements and 0.1 mg/mL gentamicin in 25 cl and 75 cl cell culture flasks (Falcon^®^, Thermo Fisher Scientific, Waltham, MA, USA). HBMEC were obtained from ScienCell, Provitro AG, Berlin, Germany) and cultured in ECM (ScienCell, Provitro AG, Berlin, Germany) containing supplements and 100 U/mL penicillin, and 100 µg/mL streptomycin on fibronectin-coated cell culture flasks (Falcon^®^, Thermo Fisher Scientific, Waltham, MA, USA). The cells were incubated at 37 °C until they reached 90% confluency. For subcultivation of HUVEC the Promocell Detach Kit was used, whereas HBMEC were detached using Trypsin/EDTA (0.025%/0.01 mM) and 10% FCS/PBS following instructions of the manufacturer. Cells were used from passages 4–6.

### 4.5. RNA Isolation and Quantitative Real-Time-PCR

HUVEC and HBMEC were cultured in ECGM2 (Promocell, Heidelberg, Germany) or ECM (ScienCell, Provitro AG, Berlin, Germany), respectively, on 6-well plates (Sarstedt^®^, Nümbrecht, Germany Newton, NC, USA) until cells reached 80% confluency. Following a 4 h starvation in 0.1% FCS in ECBM2 (Promocell, Heidelberg, Germany) or 0.1% FCS in ECM, respectively, cells were stimulated with 5 ng and 25 ng of VEGF_165_ (BioLegend, San Diego, CA, USA, 583704), CXCL2 (BioLegend, San Diego, CA, USA, 582004) or IL8 (BioLegend, San Diego, CA, USA, 574204) for 24 h. Cells were detached using cell scrapers (Corning^®^, Corning, NY, USA) after application of 300 µL lysis buffer with 1% 2-mercaptoethanol per well. RNA isolation of HUVEC and HBMEC was performed by using PureLink RNA Mini Kit (Invitrogen, Carlsbad, CA, USA) according to the corresponding protocol. RNA amount was measured with a plate photometer (Infinite M200, Tecan, Männedorf, Switzerland) and quality was tested by Agilent 2100 Bioanalyzer. The cDNA synthesis was carried out by using of PrimeScript^TM^ RT reagent Kit with gDNA Eraser (TaKaRa, Saint-Germain-en-Laye, France) as described in the manufacturer’s instructions. Received cDNA was measured by photometer to determine quantity. Quantitative Real-time-PCRs (qRT-PCRs) were executed for *VEGFR1* (forward primer: CAGGCCCAGTTTCTGCCATT, reverse primer: TTCCAGCTCAGCGTGGTCGTA), *VEGFR2* (forward primer: CATGTACGGTCTATGCCATTCCTC, reverse primer: TTGGCGCACTCTTCCTCCAAC), *CXCR1* (forward primer: GCAGCTCCTACTGTTGGACA, reverse primer: GCCCTACCCCACAGAAAGTC) and *CXCR2* (forward primer: GGTGTCCTACAGGTGAAAAG, reverse primer: TGTCACTCTCCATGTTAAAA) using triplicates in a 10 µL reaction volume and the TB GreenTM Premix Ex TaqTM Kit (TaKaRa, Saint-Germain-en-Laye, France). 18S (forward primer: GGCCCTGTAATTGGAATGAGTC, reverse primer: CCAAGATCCAACTACGAGCTT [67]) was used as reference gene. If not indicated otherwise primer sequences were designed with Primer BLAST by the National Center for Biotechnology Information, U.S. National Library of Medicine and purchased from TIB MOLBIOL, Berlin, Germany. qRT-PCRs were performed with the Quant Studio 6 Flex System (Thermo Scientific). Target mRNA was normalized to expression of 18S. Relative quantification method (ΔCt) was used for analyses.

### 4.6. Cell Proliferation Assay

The CyQUANT proliferation assay with HUVEC (Promocell, Heidelberg, Germany) was performed according to the manufacturer’s protocol (CyQUANT Direct Cell Proliferation Assay, Life Technologies, Carlsbad, CA, USA).

16,000 HUVEC cells were seeded per well in a 96-well flat-bottomed µ-clear black plate (Greiner Bio-One, Kremsmünster, Austria) in ECGM2 media (Promocell, Heidelberg, Germany) and grew for 24 h. The cells were starved overnight in ECGM2 medium with 0.1% FCS but without growth factors. Afterwards, the recombinant human proteins VEGF_165_ (BioLegend, 583704), CXCL2 (BioLegend, 582004), IL8 (BioLegend, 574204) were added in starvation medium to the cells at different concentrations. After 24 h CyQUANT reagent was added to the cells for 1 h.

Images of cells were taken and analyzed by ImageJ 1.53c, or the relative fluorescence intensity was measured using a multi-well spectrophotometer (Tecan, Männedorf, Switzerland) at the excitation wavelength of 485 nm and the emission wavelength of 525 nm.

### 4.7. Boyden Chamber Cell Migration Assay

Cell migration was accessed via the Boyden chamber assay using Fluoroblock cell culture inserts (BD Falcon^®^, Thermo Fisher Scientific, Waltham, MA, USA) with 8 µm pore size and 24-well companion plates. HUVEC cells were starved in ECGM2 media with 0.1% BSA (Sigma, St. Louis, MO, USA) for 4 h. The inserts were coated with 10 µg/mL Fibronectin (Merck/Millipore, Darmstadt, Germany) and 5 × 10^4^ cells diluted in starvation medium were seeded on the top of each insert. VEGF_165_, IL8 and CXCL2 in starvation medium were added at different concentrations to the lower chamber. HUVEC migrated for 16 h, fixed with methanol for 5 min and stained with DAPI for another 5 min. Four pictures were taken from each membrane using an inverse fluorescence microscope (Zeiss Axio Observer Z1, Jena Germany). The images were analyzed with ImageJ 1.53c software.

### 4.8. Angiogenesis Assay

Spheroids were generated using 1 mL 0.6% or 1.2% Methocel and 4 mL ECBM2 media (Promocell, Heidelberg, Germany) or ECM (ScienCell, Provitro AG, Berlin, Germany) containing 0.1% FCS and 20,000 HUVEC cells/mL or 40,000 HBMEC cells/mL (ScienCell, Provitro AG, Berlin, Germany), respectively. 120 × 25 µL of the cell suspension were plated on square Petri dishes and incubated as hanging drops. After 24 h, the spheroids were harvested using 5 mL of 10% FCS/PBS solution, and centrifuged for 3 min at 500× *g*. Spheroids were embedded in a 1.5 mg/mL Collagen I (Ibidi, Gräfelfing, Germany, 50202) gel in 8-well glass bottom chamber slides (Sarstedt, Nümbrecht, Germany). After polymerization, 200 µL of ECBM2 or ECM containing 0.1% FCS and molecules (25/50/100 ng/mL; VEGF, CXCL2, IL8) or CXCR2-antagonist were added as indicated. SB225002 (Tocris, Bristol, UK) was dissolved in 100 mM DMSO and diluted in the respective media to a final concentration of 0.03, 0.06 and 0.125 µM. Spheroids were incubated for 24 h, and then analyzed using a confocal microscope (Nikon A1Rsi+, Düsseldorf, Germany). The trainable WEKA segmentation tool in ImageJ [68] was used to classify the spheroids. For the final analysis of the sprouting area and sprout length, binary images of the core and sprouts were generated from the classified images.

### 4.9. µ−Slide-Chemotaxis Assay

Chemotaxis µ-Slide (Ibidi, Gräfelfing, Germany), special beveled pipette tips (Greiner Bio-One) as well as ECBM2 containing 0.1% FCS were pre-equilibrated in a 37 °C humidified incubator for 24 h. HUVEC were cultured and resuspended at 3 × 10^5^ cells/100 μL in ECBM2 containing 0.1% FCS. Cells were seeded and cultivated according to the manufacturer’s protocol. After 1 h, cell attachment was validated through microscopy. The reservoirs on either side of the observation chamber were slowly filled with 65 µL chemoattractant-free medium using beveled pipette tips. Immediately before transferring the chemotaxis µSlides to the microscope, the chemoattractant (10 ng/mL VEGF, CXCL2) and the CXCR2 antagonist SB225002 (0.03 µM, Tocris) were added to the reservoirs. The µSlides were secured in position on a controlled motorized stage in a temperature controlled (37 °C), humidified heat chamber surrounding the Nikon Widefield Ti2 microscope. Time-lapse images were taken every 10 min at 10× magnification for up to 18 h with the sCMOS, PCO.edge camera. The images were exported as multipage TIFF files. 50–60 cells per observation area were selected randomly and tracked individually in random order using the ImageJ 1.53c “Manual Tracking” plug-in. Cells which divided during the experiment were not used for further analysis. “Chemotaxis and Migration tool” (Ibidi, Gräfelfing, Germany) was used to quantify various chemotactic and chemokinetic responses such as the trajectory plots after 6, 12 and 15 h, the accumulated distance (total cell path traveled over time), the Euclidean distance (the shortest distance between start and end points), the displacement of the center of mass (average end position of tracked cells), the forward migration index (FMI; the ratio between the net distance traveled on the relevant axis and the accumulated distance), and the cell velocity after 6, 12, and 15 h.

### 4.10. Statistics

Statistical analyses were performed using GraphPad Prism Software (San Diego, CA, USA), displayed as mean ± standard deviation (SD). Comparisons between groups were carried out by one-way ANOVA with Bonferroni correction or two-tailed unpaired or paired Student’s *t*-test as indicated. Survival data were compared using the Log-rank test to determine significance. Pearson’s correlation coefficient was used to determine the association between expression levels of different genes. *p* < 0.05 was defined as statistically significant.

## 5. Conclusions

We showed that the angiogenic capacity of the CXCL2/IL8/CXCR2 pathway as well as the remarkable impact of IL8 and CXCL2 on human endothelial cells were comparable to VEGF. These molecules could be major targets to overcome the therapy resistance against anti-angiogenic approaches using VEGF/VEGFR pathway inhibitors. Clinical relevance was highlighted by demonstrating the overexpression of the alternative proangiogenic molecules in patient samples of glioblastoma and their correlation with worse overall survival. However, further studies are warranted to fully understand the impact of the CXCL2/IL8/CXCR2 axis on glioblastoma as well as the possible role of interference with this signaling in future therapy approaches.

## Figures and Tables

**Table 1:** Patients’ characteristics.

Age in Years (mean ± SD)	51.50 ± 19.22
GBM	57.86 ± 18.20
EP	38.75 ± 16.03
**Gender (f/m)**	**8/4**
GBM	5/3
EP	3/1
**Localization of the tumor**	
frontal	1
parietal	1
temporal	3
occipital	1
operculum	2
**MGMT status**	
positive/negative/n.a.	3/4/1
**IDH**	
positive/negative/n.a.	6/1/1
**Recurrence**	
Primary/Relapse	6/2

GBM = glioblastoma, EP = epilepsy, f = female, m = male, MGMT = O-6-methylguanin-DNA-methyltransferase, IDH = isocitrate dehydrogenase, n.a. = not assigned.

## Data Availability

The datasets used and analyzed during the current study are available from the corresponding author (P.V.) upon request.
